# Neural Translation and Automated Recognition of ICD-10 Medical Entities From Natural Language: Model Development and Performance Assessment

**DOI:** 10.2196/26353

**Published:** 2022-04-11

**Authors:** Louis Falissard, Claire Morgand, Walid Ghosn, Claire Imbaud, Karim Bounebache, Grégoire Rey

**Affiliations:** 1 Centre for Epidemiology on Medical Causes of Death Inserm Le Kremlin Bicêtre France

**Keywords:** machine learning, deep learning, machine translation, mortality statistics, automated medical entity recognition, ICD-10 coding

## Abstract

**Background:**

The recognition of medical entities from natural language is a ubiquitous problem in the medical field, with applications ranging from medical coding to the analysis of electronic health data for public health. It is, however, a complex task usually requiring human expert intervention, thus making it expansive and time-consuming. Recent advances in artificial intelligence, specifically the rise of deep learning methods, have enabled computers to make efficient decisions on a number of complex problems, with the notable example of neural sequence models and their powerful applications in natural language processing. However, they require a considerable amount of data to learn from, which is typically their main limiting factor. The Centre for Epidemiology on Medical Causes of Death (CépiDc) stores an exhaustive database of death certificates at the French national scale, amounting to several millions of natural language examples provided with their associated human-coded medical entities available to the machine learning practitioner.

**Objective:**

The aim of this paper was to investigate the application of deep neural sequence models to the problem of medical entity recognition from natural language.

**Methods:**

The investigated data set included every French death certificate from 2011 to 2016. These certificates contain information such as the subject’s age, the subject’s gender, and the chain of events leading to his or her death, both in French and encoded as International Statistical Classification of Diseases and Related Health Problems, Tenth Revision (ICD-10) medical entities, for a total of around 3 million observations in the data set. The task of automatically recognizing ICD-10 medical entities from the French natural language–based chain of events leading to death was then formulated as a type of predictive modeling problem known as a sequence-to-sequence modeling problem. A deep neural network–based model, known as the Transformer, was then slightly adapted and fit to the data set. Its performance was then assessed on an external data set and compared to the current state-of-the-art approach. CIs for derived measurements were estimated via bootstrapping.

**Results:**

The proposed approach resulted in an F-measure value of 0.952 (95% CI 0.946-0.957), which constitutes a significant improvement over the current state-of-the-art approach and its previously reported F-measure value of 0.825 as assessed on a comparable data set. Such an improvement makes possible a whole field of new applications, from nosologist-level automated coding to temporal harmonization of death statistics.

**Conclusions:**

This paper shows that a deep artificial neural network can directly learn from voluminous data sets in order to identify complex relationships between natural language and medical entities, without any explicit prior knowledge. Although not entirely free from mistakes, the derived model constitutes a powerful tool for automated coding of medical entities from medical language with promising potential applications.

## Introduction

### Background

The democratization of electronic health record databases has created countless opportunities to gain precious insights in fields ranging from precision medicine to public health and epidemiology. However, these databases still present many challenges, both technical and methodological, that make their exploitation cumbersome. As an example, natural language is extensively present in some health-related databases, while being notoriously difficult to handle with traditional statistical methods and preventing most international comparisons due to language barriers. In order to counter these undesirable properties, several approaches have been devised. For instance, by encapsulating most medical entities in a standardized hierarchical tree structure, the International Statistical Classification of Diseases and Related Health Problems, Tenth Revision (ICD-10) [1] offers a powerful and expressive way of organizing analytics-compatible health databases. On the other hand, ICD-10 entities are significantly less intuitive for human users than natural language and require years of training and practice to handle fluently. As a consequence, the data production of classification-based medical data is usually handmade, expansive, and time-consuming. Several attempts have been made to design artificial intelligence–based systems that are able to automatically derive medical entities from natural language, some with quite promising performance [2-4]. However, all of them fall short in automating the complex production schemes inherent to medical databases, specifically in regard to their high data-quality standards.

However, recent innovations in deep artificial neural networks have achieved significant progress in natural language processing (NLP) [5,6]. In particular, their applications in the field of machine translation [7-9], fueled by increases in both data and computing power, repeatedly bring automated systems closer and closer to human-level performance. Several attempts have been made to apply these powerful techniques in an electronic health database setting, most of them with mitigated success. As an example, the current state of the art in ICD-10 entity recognition from natural language in death certificates still remains a combination of expert systems and support vector machine (SVM)–based classical machine learning [2]. Several explanations exist for this discrepancy between traditional machine translation and medical entity recognition. First, deep artificial neural network–based methods are known to require huge amounts of data for optimal performance. However, most experiments were either performed with slightly out-of-date neural architectures or with data set sizes at least an order of magnitude below what would be typically required [10]. On the other hand, the Centre for Epidemiology on Medical Causes of Death (CépiDc) has been storing French death certificates at the national scale since 2011 in both natural language and ICD-10–converted formats. The entire database amounts to just under 3 million death certificates, thus providing considerably better settings in which to investigate the potential applications of deep neural networks in medical entity recognition.

This paper formulates the process of ICD-10 entity recognition from natural language as a sequence-to-sequence (Seq2Seq) statistical modeling problem and proposes to solve it with a variation one of the state-of-the-art machine translation neural architectures, the Transformer. The Methods section focuses on describing the aforementioned statistical modeling problem and overall methodology. The Results section reports the results of the experiments that were performed on the French CépiDc data set as well as a comparison with the current state of the art. The Discussion section presents a discussion on the model’s potential limitations through an error analysis and describes potential elements for improvement.

### Related Work

The task of identifying ICD-10 medical entities from natural language, whether in French or in any other language, is a well-investigated problem, where several promising approaches have already been proposed. Most of these solutions were published at the Conference and Labs of the Evaluation Forum (CLEF) eHealth challenge [2,3,10], a competition held annually where teams compete to solve NLP tasks on medical textual data. For instance, the task of recognizing ICD-10 entities from death certificates, in several languages including French, have been addressed several times over the years in this competition. So far, when it comes to the task of extracting ICD-10 entities from French death certificates, the state of the art is held by the Laboratoire d'Informatique pour la Mécanique et les Sciences de l'Ingénieur (LIMSI); they used a hybrid approach that combined data-based dictionaries for feature engineering and linear SVMs. However, nowadays, most NLP tasks are typically better handled by neural network–based architectures. These deep learning–based approaches have been applied to the problem at hand in this paper, mainly through a range of Seq2Seq architectures, as follows:

- Recurrent neural network–based encoder-decoder architectures, either with or without attention [11]
- Convolutional neural network–based encoder-decoder architectures [12,13]
- Fully attentional, although pretrained, architectures using a Bidirectional Encoder Representations from Transformers (BERT) model and transfer learning [14,15].

However, all those techniques, at least when applied to French data, failed to outperform the LIMSI’s feature engineering–based approach. A possible explanation for this observation might lie in the data set that the teams were given. Indeed, their sample sizes were generally less than 200,000 observations [2]; this is usually far from enough for proper training of advanced deep learning models, as modern neural architectures in the neural translation academic literature usually train on data sets with up to tens of millions of observations [9]. This might also explain why teams using fully attentional models, which are the current state-of-the-art models in neural translation, used pretrained architectures and transfer learning with BERT instead of training a full neural architecture end to end in a purely supervised fashion. The latter is exactly what this paper sets out to investigate and constitutes, at least to the authors’ knowledge, the first attempt at training a modern, fully attentional, end-to-end trained model on a data set with a sample size compliant with the requirements of modern deep learning methods.

## Methods

### Ethical Considerations

The use of the mortality data investigated in this paper aligns with the mission of Inserm to produce national statistics on the medical causes of death, as listed in Article L2223-42 of the general code of local authorities (Code général des collectivités territoriales), after consulting the French National Commission for Data Protection and Liberties (Commission Nationale de l'Informatique et des Libertés).

### Materials

#### Overview

The data set used for this study consists of every available death certificate found in the CépiDc database for the years 2011 to 2016, representing just under 3 million training examples. These documents record various types of information about their subjects, including the chain of events leading to the subject’s death, written by a medical practitioner.

#### Causal Chain of Death

The causal chain of death constitutes the main source of information available on a death certificate in order to devise mortality statistics. It typically sums up the sequence of events that led to the subject’s death, starting from immediate causes, such as cardiac arrest, and progressively expanding into the individual’s past and to the underlying causes of death. The World Health Organization (WHO) provides countries with a standardized causal chain of events format, which France follows, alongside most developed countries. This WHO standard asks the medical practitioner in charge of reporting the events leading to the subject’s passing to fill out a two-part form in natural language. The first part is comprised of four lines, in which the practitioner is asked to report the chain of events in inverse causal order (ie, immediate causes are reported on the first lines, and underlying causes are reported on the last lines). Although four lines are available for reporting, they do not all need to be filled. In fact, the last available lines are rarely used by the practitioner. The second part is comprised of two lines in which the practitioner is asked to report “any other significant conditions contributing to death but not related to the disease or condition causing it” [16] that the subject may have been suffering from.

In order to counter the language-dependent variability of death certificates across countries, a preprocessing step is typically applied to the causal chain of events leading to the individual’s death, where each natural language–based line on the certificate is converted into a sequence of codes defined by the ICD-10 [1]. The ICD-10 is a medical classification created by the WHO that defines 14,199 medical entities (eg, diseases, signs, and symptoms) distributed over 22 chapters; entities are encoded with three or four alphanumeric decimal symbols (ie, one letter and two or three digits), 5615 of which are present in the investigated data set. Table 1 shows an example of a causal chain of events, taken from an American death certificate, in both natural language and ICD-10 formats.

**Table 1:** Example of a causal chain of events leading to death as written in natural language and as ICD-10 codes.

Part of form		Natural language	ICD-10^a,b^ encoding
**Part 1**			
	Line 1	Stroke in September left hemiparesis	I64 G819
	Line 2	Fall scalp laceration fracture humerus	S010 W19 S423
	Line 3	Coronary artery disease	I251
	Line 4	Acute intracranial hemorrhage	I629
Part 2		Dementia depression hypertension	F03 F329 I10

^a^ICD-10: International Statistical Classification of Diseases and Related Health Problems, Tenth Revision.

^b^Some natural language lines correspond to several ICD-10 codes, whose orders matter in the overall coding process.

As previously mentioned, the process of converting the natural language–based causal chain of events leading to death into an ICD-10 format is the main focus of this paper. Consequently, the latter will be selected as the target variable and the former as the main explanatory variable for the neural network–based predictive model that will be further defined.

For reasons related to the underlying cause of death production process, the natural language–based chain of events and its ICD-10–encoded counterpart suffer from alignment errors at the line level, as shown in Table 2. Although qualitatively deemed quite rare, this misalignment phenomenon brings sufficient noise into the data set to prevent model convergence while fitting models with line-level sentence pairs.

**Table 2:** Death certificate from showcasing the misalignment phenomenon.

Part of form		Natural language	ICD-10^a^ encoding
**Part 1**			
	Line 1	Stroke in September left hemiparesis	I64 G819
	Line 2	Fall scalp laceration fracture humerus	S010 W19 S423
	Line 3	Coronary artery disease	I629^b^ I251
	Line 4	Acute intracranial hemorrhage^b^	N/A^c^
Part 2		Dementia depression hypertension	F03 F329 I10

^a^ICD-10: International Statistical Classification of Diseases and Related Health Problems, Tenth Revision.

^b^The ICD-10 code related to line 4 has been moved to line 3 by a human coder. Concatenating lines in a backward fashion restores alignment while preserving ordering.

^c^N/A: not applicable; the code that was previously here was moved to line 3, leaving this line blank.

**Figure 1:**
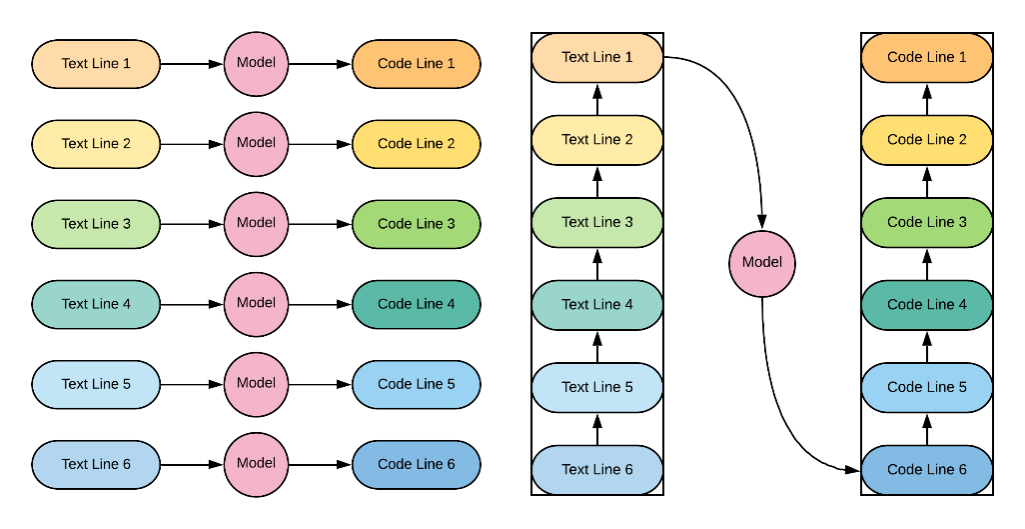
The original modeling problem and the modified investigated problem. In the original modeling problem (left), each certificate line is taken as an input variable to predict its corresponding ICD-10 code line. In the modified investigated problem (right), all certificate lines are concatenated and taken as an input variable to predict the corresponding concatenated ICD-10 code line. Lines 1-5 are from part 1 of the death certificate, and line 6 is part 2 of the certificate. ICD-10: International Statistical Classification of Diseases and Related Health Problems, Tenth Revision.

In order to bypass this critical flaw in the investigated data set, a decision was taken to consider as input and target variables the certificate lines concatenated in a backward fashion (from part 2 to line 1 in part 1), as can be seen in Figure 1. This slight change in data format does not significantly alter the problem at hand, as the investigated model is still trained to recognize ICD-10–encoded medical entities from natural language. If anything, the modified modeling problem can be expected to be more difficult, as both the variance and dimensionality of both input and target variables have increased. Several methods are available to retrieve line-level aligned predictions from a model trained in such a configuration, for instance, using a combination of transfer learning and pruned tree search.

#### Miscellaneous Variables

From gender to place of birth, a death certificate contains various additional types of information on its subject besides the chain of events leading to death. As some of these items are typically used by both expert systems and human coders to detect ICD-10 entities in the chain of events, they present an interest as explanatory variables for the investigated predictive model. After consultation with expert coders, the following items available on French death certificates were selected as additional exogenous variables:

- Gender (two-state categorical variable)
- Year of death (six-state categorical variable)
- Age, categorized into 5-year intervals, with the exception of subjects less than 1 year of age, who were divided into two classes depending on whether they were more than 28 days of age
- Origin of the death certificate (two-state categorical variable, from either the electronic- or paper-based death certification pipeline).

Strictly speaking, the subject’s year of passing should only have a limited effect on the relationship between natural language and its contained medical entities. However, the WHO-defined coding rules, as well as their interpretations by human coders, evolve slightly over the years. As a consequence, the model should benefit, in terms of predictive performance, from being able to differentiate between different years.

Similarly, the impact of the certificate’s origin on the model’s predictive power is not entirely obvious at first sight. However, the data entry process for the paper-based certificates is handled by humans through speech recognition technology. In addition, the data entry clerks are asked to apply a small set of normalization rules to the natural language. Electronic death certificates, however, are received directly from the medical practitioner as is. As a consequence, distribution shifts are to be expected from the paper- to electronic-based chain of events, and including this information as an explanatory variable might be beneficial for the model’s predictive power.

### Model Definition

With both the explanatory and target variables well defined, the investigated modeling problem can be defined as follows:



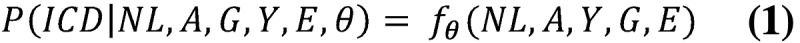



The elements of equation 1 are defined as follows:

- *P(X)*) is the probability density of discrete random variable *X*
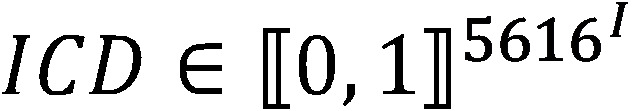
 is the sequence of ICD-10 codes present on the death certificate concatenated on a single line of sequence length *I*
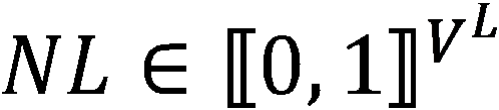
 is the line in natural language, tokenized with a vocabulary *V* and of sequence length *L*
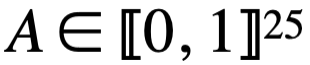
 is the categorized age
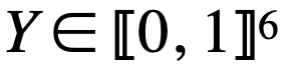
 is the year of death
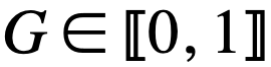
 is the gender
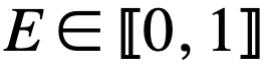
 is the death certificate’s origin
- *f*_θ_ is a mapping from the problem’s input space to its output space, parameterized in θ∈*R*^n^, a real-valued vector (typically a neural network) of dimensionality *n*∈N, the model’s dimensionality.

Theoretically, the derived modeling problem is typical of traditional statistical modeling problems and could be solved using multinomial logistic regression. In practice, however, this approach presents a significant drawback. In this setting, the investigated target variable constitutes a categorical variable with 5616^20^ distinct states—death certificates in the data set have, at most, 20 ICD-10 codes in them, each of which can take 5616 distinct values—thus rendering the analysis intractable, both in terms of computational expanses and sample size requirements. This type of approach, however, makes no use of the data’s inherent sequential nature, which allows the rewriting of the investigated modeling problem as follows:



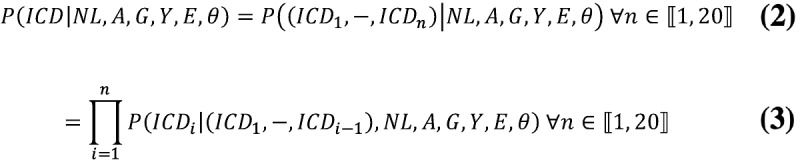



where 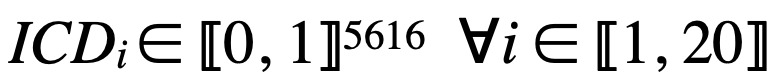 is the *i*-th code present on the code line.

Factors in the right-hand side of equation 2 can be interpreted as constituting a distinct predictive modeling problem, all with an output variable distributed across all ICD-10 codes. Although still highly dimensional, predicting output variables of such dimensionality is typically tractable with modern machine learning techniques [7]. However, they present two significant drawbacks for traditional modeling techniques: (1) the number of output variables to predict varies across observations in the data set (not all death certificates have 20 ICD-10 codes) and (2) the output variables’ distributions are conditioned on previous ones.

This particular formulation is known in the deep artificial neural network community as a Seq2Seq modeling problem [7] and has been an active area of research for the past few years. As one of the state-of-the-art neural architectures devised in the field, the Transformer [9] was chosen as the predictive model investigated in the following experiments. It was recently outperformed by the Evolved Transformer [17], a variation on the former. However, both approaches were investigated and yielded similar results. The Transformer architecture was retained due to its availability of official and maintained implementations, and the final results further displayed were obtained using an ensemble of seven such models. Each of the ensemble models’ hyper-parameters and individual performances are available in Tables S1 and S2 of Multimedia Appendix 1, respectively.

Several specificities in the previously defined modeling problem required small adaptations to the Transformer architecture. However, the authors feel that their complexity falls outside the scope of this paper. The interested reader will, however, find a complete description of these modifications as well as a visualization in Figure S1 of Multimedia Appendix 1.

Finally, the authors are aware that many other approaches to sequential learning architectures are available, and have already been used, in order to address the problem investigated in this paper. The current state of the art on French death certificates, for instance, uses a multi-label classification approach. The authors chose not to investigate those methods for several reasons.

First, the task of extracting ICD-10 codes from natural language on death certificates is only a preliminary step in the production of a mortality statistics pipeline. The final task in this process is to derive the underlying cause of death, from these ICD-10 codes, following a set of rules defined by the WHO. The choice of the underlying cause of death from this set of rules heavily depends on the codes’ order in the certificate. As a consequence, it is of paramount importance that the model be able to output these codes in the proper order, which is simply unachievable with a multiclass classification approach; this makes the problem a sequential learning problem, as our output is, indeed, a sequence of variable-length tags taken from a set of well-defined classes. However, several approaches other than Seq2Seq are still available to solve such problems, such as connectionist temporal classification, which is typically used in optical character recognition tasks.

Second, the ICD-10 codes that the model needs to output are not necessarily independent. For instance, the presence of a given code in the outputted sequence can significantly alter other codes present in the sequence. As an example given by our expert coder, hematoma-related codes can be found in two ICD-10 chapters: first in chapter 9 of the ICD-10 classification (ie, codes related to circulatory diseases, beginning with an “I”) and then in chapter 19 (ie, codes related to injury, poisoning, and certain other consequences of external causes, beginning with an “S” or a “T”). The choice of attributing the presence of the entity “hematoma” on a death certificate to the first or second possible chapter depends on whether an external cause—meaning an ICD-10 code from chapter 20—has already been outputted previously while converting the death certificate into codes. In order to account for such dependencies, we are compelled to model the joint distribution of the output sequence conditioned on the input variables, which is exactly what Seq2Seq is about. Therefore, the choice of using Seq2Seq approaches to solve the modeling problem investigated in this paper becomes not only natural but almost compulsory. In addition, due to the data-driven tokenization used in order to make use of the ICD-10 classification’s hierarchical nature, some tokens that the model is allowed to predict are not valid ICD-10 codes. For instance, the code “I659” could be decomposed into a sequence of two codes (ie, “I65” and “9-” with the “-” character at the end used to keep track of spaces between codes). It appears clear here that when the model needs to output an “I659” code, predicting “9-” in itself is not possible without any conditioning on “I65” appearing earlier.

### Training and Evaluation Methodology

The investigated model was trained using all French death certificates from the year 2011 to 2016. A total of 5000 certificates were randomly excluded from each year; these were distributed into a validation set for hyper-parameter fine-tuning and into a test data set for unbiased prediction performance estimation (2500 certificates each), resulting in three data sets with following sample sizes: (1) training data set (3,240,109 records), (2) validation data set (30,000 records), and (3) test data set (30,000 records).

The model was adapted from TensorFlow’s official Transformer implementation; TensorFlow is a Python-based distributed machine learning framework. Training was performed on three NVIDIA RTX 2070 GPUs simultaneously with a mirrored distribution strategy using a variant of stochastic gradient descent, the Adam optimization algorithm.

Hyper-parameters were first initialized following the Transformer’s base setting, according to the architecture’s authors. Further fine-tuning of a selected number of hyper-parameters was performed using a random search guided on the validation set. The interested reader will find a complete description of the training process and hyper-parameter values defining this model in Multimedia Appendix 1.

After training, the model’s predictive performance was assessed on the test data set, which was excluded prior to training, as mentioned earlier, and compared to the current state of the art, obtained by the LIMSI during the 2017 CLEF eHealth challenge [2]. As the CLEF eHealth challenge only provided electronic certificates to the contestants, and in order to ensure comparability, the model’s performance was assessed using paper-based and electronic certificates, separately. For the same reason, the performance metrics used for model evaluation were selected as follows:



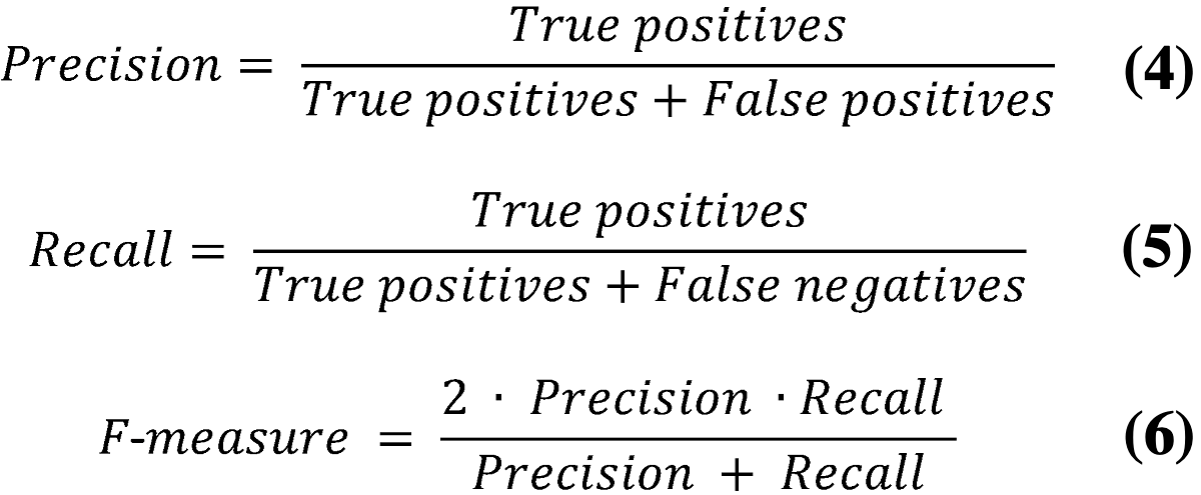



The elements of equations 4 and 5 are defined as follows:

- True positives: the number of codes predicted by the model that are present in the test set’s true output target
- False positives: the number of codes predicted by the model that are not present in the test set’s true output target
- False negatives: the number of codes not predicted by the model that are present in the test set’s true output.

Note that predictions are considered as true positives only for exact code matches, up to the fourth character. Table 3 shows an example of how this can affect the reported performance, by focusing on a line of the causal chain of events leading to death reported in Table 1 and fictional examples of predictions, as follows:

- The first prediction example outputs two incorrect codes. The number of true positives is, thus, 0, leading to all metrics being evaluated as 0.
- The second prediction example correctly outputs the first code (I64: “Stroke”) but fails to correctly output the second code’s fourth character (G81: “Hemiplegia” is predicted instead of the ground-truth value G819: “Hemiplegia, unspecified”). Although the prediction and ground truth are quite similar (ie, they share the three first characters), this code is considered incorrect, which leads to counts of both one false positive (ie, the code was predicted incorrectly) and one false negative (ie, the correct G819 code was not predicted), leading to all metrics being evaluated as 0.5.
- The third prediction example correctly outputs the first code but fails to recognize any additional codes from the textual input, leading to a precision of 1 (ie, all predicted codes are indeed true positives) and a recall of 0.5 (ie, one code present in the ground truth was not predicted). This then leads to an F-measure of 0.66. Note that in this context, the F-measure is higher than in the second example.
- The fourth prediction example correctly outputs both codes but also outputs two additional and completely unrelated codes, leading to a precision of 0.5 (ie, only half of the predicted codes are present in the ground truth) and a recall of 1 (ie, all codes present in the ground truth were correctly predicted), leading to an F-measure of 0.66.
- The fifth prediction example correctly outputs both codes and does not predict any additional codes (ie, perfect prediction), leading to all metrics being evaluated as 1.
- The sixth prediction example correctly outputs both codes and does not predict any additional codes. However, the codes are in the wrong order, but this is not penalized in any way in the metrics definitions, so this prediction is associated with metrics all being evaluated as 1.

**Table 3:** Examples of how the selected performance metrics behave for different predictions. The input text was “stroke in September left hemiparesis” and the true ICD-10 encoding was I64 and G819.

Prediction example	ICD-10^a^ codes	Precision	Recall	F-measure
1	B189 H155	0.0	0.0	0.0
2	I64 G81	0.5	0.5	0.5
3	I64	1.0	0.5	0.66
4	I64 G819 A338 B87	0.5	1.0	0.66
5	I64 G819	1.0	1.0	1.0
6	G819 I64	1.0	1.0	1.0

^a^ICD-10: International Statistical Classification of Diseases and Related Health Problems, Tenth Revision.

The informed reader might find that these metrics stray away from common machine translation system benchmarking metrics, such as bilingual evaluation understudy (BLEU) or negative log perplexity scores [7-9,18], but the former were the only ones used in comparable work. As BLEU and negative log perplexity have close to no absolute interpretability without comparisons to alternative methods, their use was discarded from the experiment. In order to present the reader with a more comprehensive view of the performance of the proposed approaches, these accuracy metrics were also derived on a per-chapter basis, again on the same test set, and 95% CIs were computed using bootstrapping.

## Results

### Performance Evaluation

The ensemble of Transformer models were trained as previously described for approximately 3 weeks; the final ensemble’s predictive performance and that of the current state-of-the-art model are reported in Table 4. As previously mentioned, the performance of the current state-of-the-art model was assessed based on electronic certificates only and should, as a consequence, be compared to the performance of the proposed approach based on a similar situation. Because paper-based certificates are still more common than their electronic counterparts in France (ie, approximately 90% of certificates in the data set are paper based), the performance of the approach using all certificates and that of the paper-based certificate approach are also displayed.

**Table 4:** Assessments of the current state-of-the-art model and the proposed approach.

Approach	F-measure (95% CI)^a^	Precision (95% CI)	Recall (95% CI)
Current state of the art: LIMSI^b^	0.825^c^	0.872^c^	0.784^c^
Proposed approach: electronic certificates	0.952 (0.946-0.957)	0.955 (0.95-0.96)	0.948 (0.943-0.954)
Proposed approach: paper-based certificates	0.942 (0.941-0.944)	0.949 (0.947-0.95)	0.936 (0.934-0.937)
Proposed approach: all certificates	0.943 (0.941-0.944)	0.949 (0.948-0.951)	0.937 (0.935-0.938)

^a^95% CIs were derived by bootstrapping.

^b^LIMSI: Laboratoire d'Informatique pour la Mécanique et les Sciences de l'Ingénieur.

^c^95% CIs were not provided in the LIMSI’s publication and are, therefore, not displayed.

The proposed approach shows an F-measure that is 73% closer to a perfect score when compared to the current state-of-the-art approach. In addition to its substantial improvement in the F-measure, the proposed approach displays significantly more balanced precision and recall scores than the LIMSI’s method: from 5% relative difference to less than 1%.

A surprising result, however, lies in the model’s lower performance based on paper-based certificates. Indeed, the standardization they receive due to their voice-based data collection process considerably reduces variance and prevents any misspelled words in the data that are potentially present in electronic-based certificates. As a consequence, model performance on the former should be expected to be higher. A potential explanation for this phenomenon lies in the potential for missing data in paper-based certificates. Indeed, when confronted with poorly written words, data clerks are allowed to replace them with the “!” symbol when the word is estimated to be unreadable; this occurs in approximately 10% of paper-based certificates. Medical coders, however, are usually more efficient in guessing the words from the written certificates, typically with the addition of contextual clues. A purely text-based approach, however, is then limited to pure guesses for those observations with missing data, logically leading to poorer performance. Because this phenomenon is absent from electronic-based certificates, it is a promising candidate for explaining this unexpected difference in performance. In addition, the model performance based on paper-based certificates that did not contain any “!” symbols in the test set led to an F-measure of 96.2%, thus providing strong evidence to support this hypothesis.

### Per-Chapter Quantitative Analysis

Although the proposed approach significantly outperformed the current state-of-the-art approach, neural network–based methods are known to present several drawbacks that can significantly limit their application in some situations. Typically, the current lack of systematic methods to interpret and understand neural network–based models and their decision processes can lead the former to perform catastrophically on incorrectly predicted cases, independent from their high predictive performance. As a consequence, the proposed model behavior in incorrectly predicted cases requires careful analysis. In addition, such an investigation can lead to significant insights that are potentially relevant when applying the derived model in practical applications.

One simple, straightforward approach to understanding the model’s weakness lies in assessing its performance on a finer-grain level, for instance, by identifying false positives and negatives not only at the global level, but per ICD-10 chapters, as can be seen in Table 5.

It appears from this table that although the most prevalent medical entities are associated with low false positive and negative rates, some rarer chapters are associated with unreasonably high error rates. Depending on their prevalence and accuracies, these chapters can be classified into two distinct categories:

1. Chapters associated with unreasonably high error rates but extremely low prevalence, such as “Diseases for the ear and mastoid process” or “Pregnancy, childbirth and the puerperium.” However, these entity groups remain rare enough within the data set to allow for alternative treatments, like manual evaluation, for instance.
2. Chapters associated with high error rates, although lower than the former, but with significant prevalence, such as “External causes of morbidity and mortality” or “Injury, poisoning and certain other consequences of external causes.”

The task of identifying these potential mistakes, however, is not entirely trivial depending on whether mistakes are of false positive or false negative types. Indeed, potential false positive errors are directly identifiable within the predicted ICD-10 code sequences. As a consequence, coding quality control for this type of mistake should be fairly straightforward to implement: one could, for instance, manually review all code sequences containing codes related to “Pregnancy, childbirth and the puerperium” systematically. Potential false negative errors, however, are inherently significantly harder to identify and require further investigation, for instance, through association rules analysis.

A number of promising leads are already available and should reasonably improve upon the proposed approach:

- Training methods adapted to imbalanced data sets, such as up-sampling or loss weighting
- Data augmentation for rare medical entities
- Addition of information to the model (ie, prenatal-related death, for instance, is explicitly defined as such on certificates)
- A hybrid approach with traditional NLP approaches, which are typically less expensive in terms of sample size requirements.

**Table 5:** False positive, false negative, and prevalence rates for each ICD-10 chapter, sorted in descending order by prevalence.

ICD-10^a^ chapter	False positives, %	False negatives, %	Prevalence, %
Diseases of the circulatory system	3.75	4.98	22.4
Symptoms, signs and abnormal clinical and laboratory findings, not elsewhere classified	3.87	4.12	21.8
Neoplasms	4.07	5.07	15.9
Diseases of the respiratory system	3.02	4.00	8.76
Endocrine, nutritional and metabolic diseases	2.17	3.44	4.83
Diseases of the nervous system	2.70	4.12	3.89
Mental, behavioral and neurodevelopmental disorders	2.88	4.14	3.58
Diseases of the digestive system	5.72	8.10	3.53
Factors influencing health status and contact with health services	19.2	19.6	3.08
Diseases of the genitourinary system	5.45	7.59	2.71
External causes of morbidity and mortality	16.6	23.5	2.57
Certain infectious and parasitic diseases	7.98	9.23	2.55
Injury, poisoning and certain other consequences of external causes	14.0	19.8	2.07
Diseases of the blood and blood-forming organs and certain disorders involving the immune mechanism	6.72	12.2	0.77
Diseases of the musculoskeletal system and connective tissue	12.2	17.3	0.62
Diseases of the skin and subcutaneous tissue	8.72	8.16	0.51
Certain conditions originating in the perinatal period	14.5	20.5	0.16
Congenital malformations, deformations and chromosomal abnormalities	22.4	25.6	0.15
Diseases of the eye and adnexa	4.93	13.6	0.076
Codes for special purposes	24.0	34.0	0.047
Diseases of the ear and mastoid process	5.60	33.3	0.017
Pregnancy, childbirth and the puerperium	50.0	33.3	0.0056

^a^ICD-10: International Statistical Classification of Diseases and Related Health Problems, Tenth Revision.

### Score Calibration Fitness Assessment

When the model is fit in a similar fashion to multinomial logistic regression, it not only yields a prediction but an associated score similar to a confidence probability. If properly calibrated, this score can offer powerful insights regarding the prediction’s quality at the individual level. Typically, a “good” score would be expected to show higher values in cases where the ICD-10 sequence is correctly predicted and lower values when incorrectly predicted. Such a well-calibrated score could, for instance, allow for real-world applications of semiautonomous systems where the following occurs:

- A threshold value for the model’s score is defined.
- All certificates whose predictions are associated with confidence scores above the threshold level are accepted without any additional human supervision.
- All certificates whose predictions are associated with confidence scores below the threshold level are systematically reviewed by a human expert and modified manually, if required.

Being able to properly filter the model’s predictions according to a well-calibrated confidence score would, thus, allow us to get the best of both worlds. Most of the certificates would be automatically coded by the autonomous system, leaving human coders with only the most complex cases.

**Figure 2:**
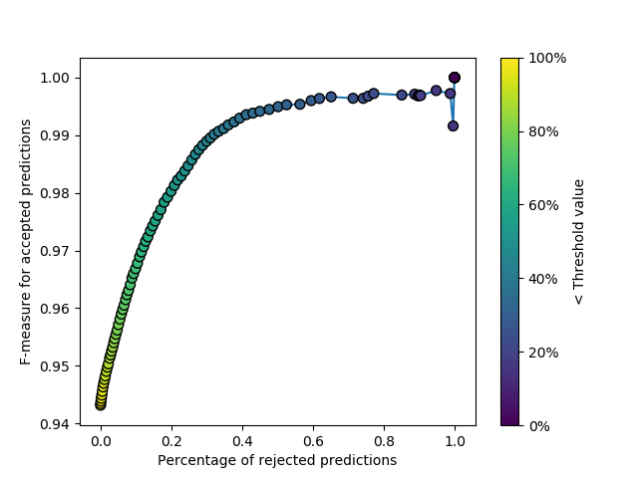
Percentage of rejected predictions versus F-measure for accepted ones. The score threshold values defining the accepted predictions are displayed as colored points.

Efficient assessment of such scores in traditional machine learning problems is typically done through visualization of receiver operating characteristic (ROC) curves. However, the sequential multinomial nature of the investigated problem renders this approach ill-defined. The plot in Figure 2, while conceptually similar to an ROC curve, was derived following a slightly different approach in order to efficiently appreciate the model score’s quality. This visualization was derived as follows:

- A grid of score threshold values was defined with a uniform grid with 0.01 intervals, corresponding to the threshold defined above, filtering between model predictions that would require human examination or not.
- For every given threshold value, we computed the percentage of predictions with inferior or equal scores, which were considered as rejected, requiring human examination due to poor score; we also computed the F-measure performance on the predictions with high enough scores that would be accepted without any human intervention following the above example.
- The percentage of accepted certificates and F-measures were plotted on a scatterplot against each other, with threshold values displayed as colored points.

By showing a clean, increasing relationship between the number of rejected predictions and the F-measure evaluated using the remaining certificates, Figure 2 strongly indicates good score calibration. As an example, by considering that only predictions associated with a confidence score lower than 0.5 do not require any additional human supervision, the system is able to code approximately 80% of all certificates present in the test set with an F-measure of 0.98, significantly higher than the value of 0.94 obtained on all test certificates.

## Discussion

### Principal Findings

The error analysis carried out so far allowed for the assessment of the model’s strengths and weaknesses on a global level. However, it failed to yield any interesting insights regarding potential model biases, for instance, toward specific coding rules. Indeed, the coding of medical entities from natural language, especially with regard to mortality statistics, is subject to a number of coding rules depending on context or pathology, with a level of specificity oftentimes reaching casuistry [1].

In addition, all results have been presented so far with a model error defined as a disagreement between the model’s output and the information contained in the database. However, building a medical database is a complex, mostly human-based process. As such, an inevitable amount of noise is to be expected in the ICD-10 codes present in the database, in two main forms. The first form is simple human errors in the ICD-10. The second form is the presence of unreadable text in paper-based certificates. Unreadable words on paper-based certificates are denoted as an exclamation point in the textual data that is fed to the model. However, human coders usually take additional time to infer these words, for instance, via queries to the medical certifier or from contextual cues. This leads to death certificates in the database where the ICD-10 sequences contain additional codes compared to the textual data available. As such, not predicting these codes would result in a drop in performance metrics, while the model has no way of predicting them. An example of such a death certificate can be found in Table S3 in Multimedia Appendix 1. These phenomena have the potential to negatively bias the proposed model’s performance estimations and should be the object of further investigation.

One straightforward, although fairly time-consuming, approach to address these two considerations can be to have an ICD-10 coding expert manually examine some of the death certificates where the model’s predictions do not match the ICD-10 codes present in the database. Two experiments were conducted following this idea.

In the first experiment, 99 certificates where the model’s predictions did not exactly match with the database’s ICD-10 variables (ie, the ICD-10 sequences differed by at least one code) were selected at random from the test set and were shown to the medical practitioner representative and final decision maker on ICD-10 mortality coding in France, who was asked to do the following with each certificate:

- Manually recode all the ICD-10 medical entities present on each death certificate by herself using the information the proposed model had access to, without access to the data set or to the model’s proposed ICD-10 sequences.
- Give a qualitative comment on the outputs of the investigated model and database as compared to hers.

Since the ICD-10 sequences derived from the medical expert and national representative for ICD-10 coding in France are significantly more reliable than the ones coming from the traditional data production process (ie, using a combination of expert system and human coders), they can be considered as exempt of any potential human error. As a consequence, comparing them to both the proposed model’s output and the ICD-10 values contained in the data set would allow for an estimation of the potential negative biases described above. This can be done, for instance, by estimating the performance metrics selected for the previous experiments, considering both the model’s predictions and the database’s values as predictions, and the medical expert’s outputs as the ground truth. Depending on the resulting values, several interpretations can be made ranging between two extreme cases:

1. If perfect agreement (ie, an F-measure of 1.0) is reached between the database’s ICD-10 sequences and the medical expert’s outputs, suggesting that the database does not have any coding mistakes, then the performance metrics reported in the Results section can safely be considered unbiased.
2. If perfect agreement is not reached between the model’s predictions of ICD-10 sequences and the medical expert’s outputs, suggesting that the model did not make any mistakes, then the performance metrics reported in the Results section should be considered significantly underestimated.

However, before estimating the performance metrics following this methodology, a slight preprocessing step is required. Indeed, on the death certificates sampled for the experiment, the F-measure estimation between the model’s prediction and the database’s ICD-10 sequences yielded a value of 0.81. This is explained by the sampling process, in which death certificates were selected where at least one code differed in both ICD-10 sequences. As a consequence, and because of the model’s performance, most ICD-10 codes present on both sequences were identical, as can be seen with the error examples presented in Tables S3 to S5 in Multimedia Appendix 1. The authors felt that this might lead to artificially high values of the estimated metrics in the experiment; consequently, we decided to delete all common codes on both the model’s outputs and the database’s values prior to metrics estimation, as shown in Table 6.

For better comparability, these statistics are reported based on both (1) certificates without missing data in the natural language–based causal chain of events leading to death (by excluding certificates containing the “!” symbol) in Table 7 and (2) all certificates in Table 8.

**Table 6:** Example of preprocessing used for the experiment on a real error example. The predicted and database ICD-10 sequences only differ by one code, while they share five codes. All shared codes were deleted from all ICD-10 sequences prior to estimation of performance metrics.

Source of ICD-10^a^ codes	ICD-10 codes before preprocessing	ICD-10 codes after preprocessing
Predicted by the model	I259 Z951 I719 C679 I10 R092	Z951
Present in the database	I259 I251 I719 C679 I10 R092	I251
Predicted by medical expert	I259 I251 I719 C679 I10 R092	I251

^a^ICD-10: International Statistical Classification of Diseases and Related Health Problems, Tenth Revision.

**Table 7:** F-measure, precision, and recall of both the database ICD-10 codes and the model’s prediction of codes compared to that of the medical expert for sampled certificates without missing data.

Source of ICD-10^a^ codes	F-measure (95% CI)	Precision (95% CI)	Recall (95% CI)
Presence in database against medical expert prediction	0.483 (0.383-0.589)	0.443 (0.341-0.555)	0.531 (0.425-0.636)
Model prediction against medical expert prediction	0.431 (0.316-0.542)	0.458 (0.338-0.580)	0.407 (0.295-0.519)

^a^ICD-10: International Statistical Classification of Diseases and Related Health Problems, Tenth Revision.

**Table 8:** F-measure, precision, and recall of both the database ICD-10 codes and the model’s prediction of codes compared to that of the medical expert for all sampled certificates.

Source of ICD-10^a^ codes	F-measure (95% CI)	Precision (95% CI)	Recall (95% CI)
Presence in database against medical expert prediction	0.613 (0.486-0.733)	0.630 (0.492-0.761)	0.596 (0.471-0.721)
Model prediction against medical expert prediction	0.370 (0.237-0.504)	0.392 (0.250-0.540)	0.351 (0.222-0.482)

^a^ICD-10: International Statistical Classification of Diseases and Related Health Problems, Tenth Revision.

Tables 7 and 8 show no significant difference in prediction performance between the proposed approach and the current data production process (ie, based on a combination of expert system and human coders), although the database’s ICD-10 values have better performance metrics in both cases. When including certificates containing missing text, the proposed model’s agreement with the medical expert increases considerably, further confirming the hypothesis that the performance metrics reported in the Results section were negatively biased.

From the qualitative comments made by the medical expert, three major types of model errors could be defined:

1. In 16% (16/99) of cases, disagreement between the current data production process and the proposed approach was due to missing information in the input text. On these specific cases, the F-measure between the model’s output and medical expert’s decision was determined to be 0.974; an example of such an error case can be seen in Table S3 in Multimedia Appendix 1.
2. In 14% (14/99) of cases, the correct ICD-10 sequence was dependent on highly contextual clues or external knowledge of world behavior (eg, someone found dead at the bottom of a set of stairs is quite likely to have suffered a fall). An example of such an error case can be seen in Table S4 in Multimedia Appendix 1.
3. In 12% (12/99) of cases, the correct ICD-10 sequence was dependent on highly nonlinear, almost casuistic rules. These were typical examples of scenarios where a hybridized deep learning and expert-based system would be beneficial; an example of such an error case can be seen in Table S5 in Multimedia Appendix 1.

The remaining cases did not elicit any comment from the medical expert.

Finally, in the second experiment, the medical expert’s ability to discriminate between human coding and the proposed approach was assessed in a Turing test-like approach. To do so, 100 additional certificates where the model’s output differed from the database’s ICD-10 sequences were sampled at random from the test set. The medical expert was shown their corresponding input features (ie, text and auxiliary variables) as well as the two ICD-10 sequences, with their provenance from either the model or the database masked, as can be seen in Table 9.

**Table 9:** Example of death certificate format given to the medical expert for the second experiment. The medical expert was asked, based on the information available in the line, to guess which of propositions 1 or 2 was produced by a human coder, with the other being the proposed model’s output.

Item	Sex^a^ of deceased	Year of death	Age of deceased (years)	Certificate text^b^	Proposition 1 (ICD-10^c^ codes)	Proposition 2 (ICD-10 codes)
Death certificate	2	2013	90	90 ans, péritonite, perforation grêle, occlusion, chirurgie digestive, infection pulmonaire, arrêt respiratoire	R54 K566 K659, K631 Y839 J958 R092	R54 K659 K631 K566 Y839 J189 R092

^a^Sex is a two-state categorical variable: 1 (female) or 2 (male).

^b^The certificate text was taken from a death certificate in France and is, therefore, written in French.

^a^ICD-10: International Statistical Classification of Diseases and Related Health Problems, Tenth Revision.

After exclusion of certificates containing missing text data, where the human coder was easily identifiable due to the apparently out-of-context additional codes (Table S3 in Multimedia Appendix 1), the medical expert was able to correctly identify the human coder in 63% (62/99; 95% CI 50.7%-73.2%) of cases, which is significantly better than random guessing, although barely.

### Conclusions

In this paper, the task of automatic recognition of ICD-10 medical entities from natural language in French was presented as a Seq2Seq modeling problem, well known in the deep artificial neural network academic literature. From this consideration, the performance of a well-known approach in the field, consisting of an ensemble of Transformer models, was investigated using the CépiDc database and was shown to reach a new state of the art. The derived model’s behavior was thoroughly assessed following different approaches in order to identify potential weaknesses and elements for improvements. Although the proposed approach significantly outperformed any other existing automated ICD-10 recognition systems based on French free text, the question of method transferability to other languages requires more investigation.

The substantial performance reported in this paper makes possible a range of promising applications in various medical-related fields, from automated medical coding to advanced natural language–based analysis for epidemiology. However, these interesting opportunities are oftentimes prohibited by these methods’ massive drawbacks, mostly their requirement for millions of annotated observations in order to perform well. Mortality data sets, despite their specificity, provide researchers with a huge amount of clean, multilingual medical text data perfectly fit for the application of deep neural networks. As a consequence, and keeping in mind the strong transfer learning capability of neural networks, the authors firmly believe that mortality data constitute one of the most promising points of entry into modern NLP methods applications in the biomedical sciences.

## Appendix

Multimedia Appendix 1 Supplementary material.

## Abbreviations

BERT: Bidirectional Encoder Representations from Transformers
BLEU: bilingual evaluation understudy
CépiDc: Centre for Epidemiology on Medical Causes of Death
CLEF: Conference and Labs of the Evaluation Forum
ICD-10: International Statistical Classification of Diseases and Related Health Problems, Tenth Revision
LIMSI: Laboratoire d'Informatique pour la Mécanique et les Sciences de l'Ingénieur
NLP: natural language processing
ROC: receiver operating characteristic
Seq2Seq: sequence-to-sequence
SVM: support vector machine
WHO: World Health Organization
