# Supplementary material for: Neural Translation and Automated Recognition of ICD-10 Medical Entities From Natural Language: Model Development and Performance Assessment
Source: JMIR Med Inform. 2022 Apr 11;10(4):e26353. doi: 10.2196/26353 (PMC9039820; doi:10.2196/26353)
Supplement: Multimedia Appendix 1 [file medinform_v10i4e26353_app1.docx]

Multimedia Appendix 1: Neural translation and automated recognition of ICD10 medical entities from natural language

# Pre-processing

## Text standardization

Minimal standardization was applied to the text data. The 6 lines present on the death certificates were concatenated with a “, ” separator, and the two following steps were applied as the only text cleaning treatments:

- All letters were put to lower case
- All space based separator were collapsed to “ “

## Tokenization for rare words

In order to reduce the problem’s dimensionality and handle rare words in the dataset, Byte pair encoding [1], the standard methodology used in the recent machine translation academic literature, was used. Its implementation used for the experiments reported in this article can be found in the official Tensorflow Transformer repository [2].

The algorithm was applied on the entire training dataset and the derived tokenization were of cardinal 500 and 2033 for the ICD10 and French corpora, respectively. In standard machine translation problems, it is usually standard procedure to tokenize both corpora with one given tokenization, in order for instance to make use of potential similar prefixes and suffixes in both languages. However, since the ICD-10 and French vocabulary are so different (the ICD-10 classification not being a natural language), the authors decided to use a distinct tokenization for each. Not that the two derived concatenation could be concatenated to get a similar result.

# Model definition

The model itself follows the traditional transformer architecture [3]. The model’s official Tensorflow implementation was used for the experiments [2]. However, the traditional Transformer model doesn’t allow for the treatment of additional conditional variables. In order to include the latter in the model, a similar methodology than that followed in [4] was chosen:

- Each element in both the ICD-10 target sequence and the French input sentences (so either an ICD-10 code or a French word) are tokenized following the tokenizations obtained using byte pair encoding. The derived tokens are then fed to a linear embedding (of dimensionality “hidden_size”, a model hyperparameter) similar to word2vec, whose parameters are randomly initialized and learned during training. A distinct embedding is created for the ICD-10 tokens and the French tokens
- Each conditional variable is also fed to a linear embedding of same dimensionality as the ICD-10 and French tokens that are learnt by the model during training. One distinct embedding is learnt per categorical variable, leading to 4 linear additional embeddings, for the gender, year, age, and origin of certificate variables.
- The mean vector of these linear projections is added in an element-wise fashion to the French embedded token sequence.
- The transformer model is used as defined in its original article on the resulting embedded sequence.


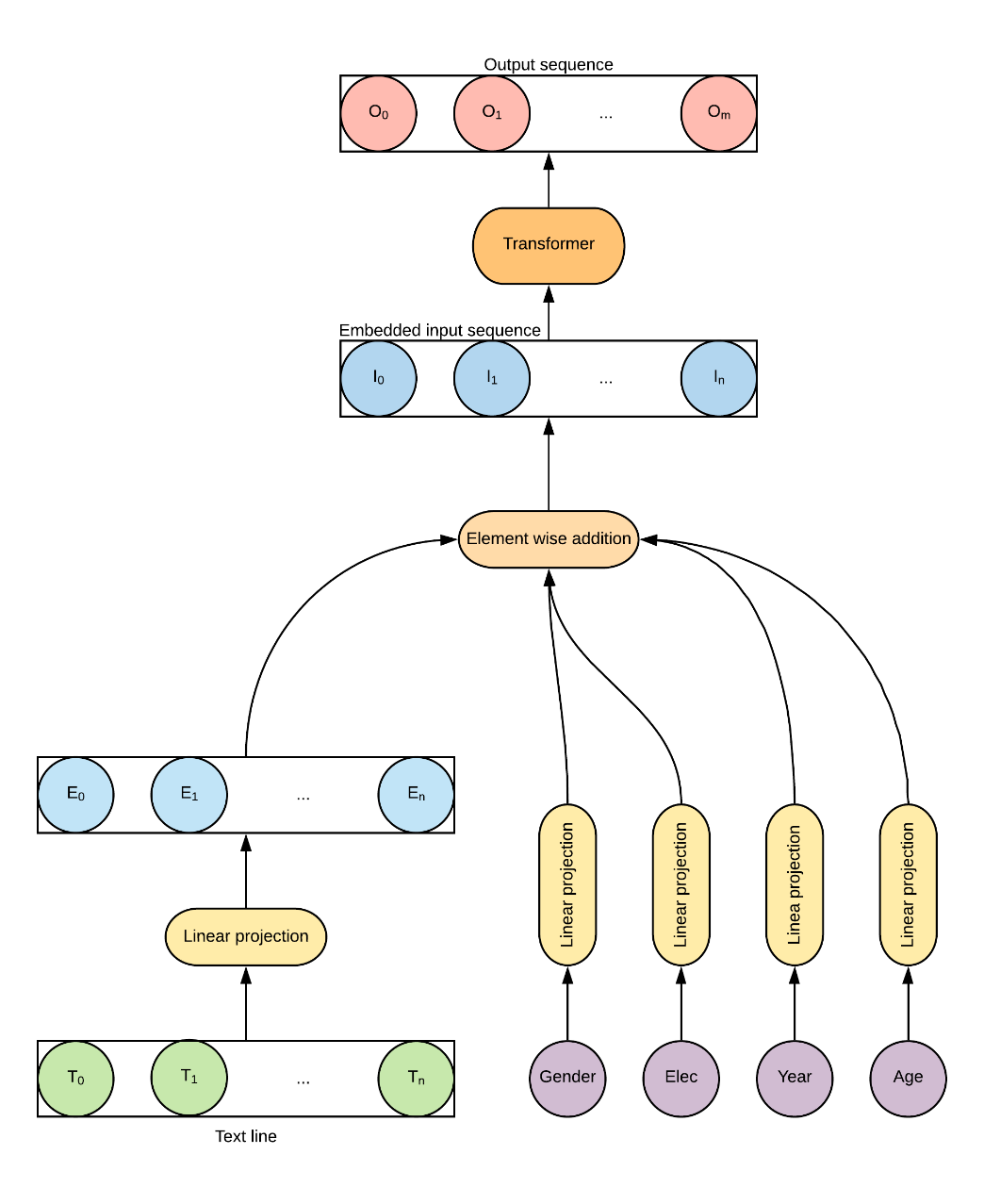


Figure S1. Transformer adaptation for the handling of conditional variables

Several approaches are available in order to incorporate the categorical variable into the Transformer architecture. The authors chose this one for several reasons:

- Element-wise addition of categorical variables into the embedded input sequence has already been performed in the past (both in independent work or by work produced by the authors [4], [5])
- The idea of element-wise addition of categorical variables to the embedded input sequence is already implicitly present in the Transformer architecture, in its positional encoding. Indeed, as the model does not use any convolutional or recurrent neural architecture, the position of each word in the sentence needs to be explicitely expressed in the input values. To do so, the authors decide to consider the position of each word in the sentence as an auxiliary categorical variable, to embed it into a “hidden_size” vector, and to add it in an element-wise fashion to each of the sentence token. They investigate whether a learnable embedding or one pre-defined is better, and end up choosing the predefined one for simplicity (this being possible because position of a token in a sentence is something reasonably easy to model using only prior knowledge). The authors followed the exact same methodology to incorporate the auxiliary conditional variables in the model, but opted for a learnable embedding, as no clear prior knowledge can be used to manually define the impact these variables have on the decision process leading to the identification of ICD-10 entities.
- The fifth prediction example correctly outputs both codes, and doesn’t predict any additional code (perfect prediction), leading to all metrics being evaluated to 1.
- The sixth prediction example correctly outputs both codes, and doesn’t predict any additional codes. The codes are however in the wrong order, but this isn’t penalized in any way in the metrics definitions, so this prediction is associated with metrics being all evaluated to 1.

# Hyperparameter search

Hyperparameter tuning was done with a random search guided by validation set’s F-measure results.

The following variable were randomly sampled from the further specified probability distributions:

- Model’s hidden size: sampled from a uniform random distribution between 256 and 512
- Batch size: For computational reasons, the batch size was defined as (100 * 512 / hidden_size)
- Learning rate: Uniformly sampled from discrete values 1. or 2. (note that this value doesn’t constitute the actual learning rate, which is modified by the function “get_learning_rate”
- Layer_postprocess_dropout: sampled from a uniform random distribution between 0 and 0.2
- Attention_dropout: sampled from a uniform random distribution between 0 and 0.2
- Relu_dropout: sampled from a uniform random distribution between 0 and 0.2

All other parameters were fixed as recommended by the BASE_MULTI_GPU settings provided in the tensorflow transformer official implementation.

40 models were trained with different hyperparameters sampled from these distributions, the best set of hyperparameter was then used to train a new set of model for ensembling.

# Ensembling method

Due to computational reasons, the traditional method for ensembling neural translation model (logits averaging during the beam search process) could not be used. The following alternative was used instead:

- Get the prediction from each model
- Compute F-measurements between all prediction candidates
- Select the prediction that shows highest F-measurements with other candidates on average

The ensemble of models was selected by a greedy search on all the models trained for the experiment (40 models trained during the hyperparameters search and additional models trained with the best hyperparameter set) guided by the F-measurement on the validation set.

The derived score was taken as the prediction scores’ average.

# Final ensemble hyperparameters

The final ensemble found by greedy exploration consisted of 7 different models, 5 of which were trained with the best set of hyperparameters revealed by the random hyperparameter searchs. The three distinct sets of hyperparameters can be found in Table S1, and the individual performances of each model can be found in Table S2.

| Hyperparameter | Set 1 (best set) | Set 2 | Set 3 |
| --- | --- | --- | --- |
| Batch size | 172 | 152 | 164 |
| Hidden size | 296 | 336 | 312 |
| Learning rate | 2. | 2. | 2. |
| Layer postprocess dropout | .073 | .12 | .005 |
| Attention dropout | .105 | .030 | .017 |
| Relu dropout | .173 | .030 | .20 |

Table S1. Sets of hyperparameters for the different models used in the final ensemble

| Model | Precision | Recall | F-measure |
| --- | --- | --- | --- |
| Model 1 (hyperparameter set 2) | 94.6 | 93.3 | 93.9 |
| Model 2 (hyperparameter set 3) | 94.5 | 93.1 | 93.8 |
| Model 3 (hyperparameter set 1) | 94.4 | 93.4 | 93.9 |
| Model 4 (hyperparameter set 1) | 94.6 | 93.4 | 94.0 |
| Model 5 (hyperparameter set 1) | 94.6 | 93.4 | 94.0 |
| Model 6 (hyperparameter set 1) | 94.6 | 93.5 | 94.0 |
| Model 7 (hyperparameter set 1) | 94.5 | 93.4 | 94.0 |
| Overall ensemble | 94.9 | 93.7 | 94.3 |

Table S2. Performance metrics on the test set for each individual model from the ensemble (on both paper and electronic certificates). The ensemble value reported in the main article is also reported to allow comparison

# Error examples

| Text | hta, insuffisance cardiaque, anévrisme aorte !, !, asystolie ! |
| --- | --- |
| Predicted ICD10 | I10 I509 I714 I500 |
| Database ICD10 | I10 I509 I714 H570 I500 R068 |
| Expert ICD10 | I10 I509 I714 I500 |

Table S3. Example of “missing data” type error. The database shows two additional codes that are not present in the text according to the medical expert. These codes are probably associated with the “!” present in the text, and were derived from a human coder reading the handwritten death certificate.

| Text | acfa, hta, connu vertige, retrouvé terre bas escalier |
| --- | --- |
| Predicted ICD10 | I48 I10 R42 R98 |
| Database ICD10 | I48 I10 R42 W10 |
| Expert ICD10 | I48 I10 R42 W10 |

Table S4. Example of contextual error. The proposed approach converts “retrouvé terre bas escalier” (which roughly translates to “found at the bottom of the stairs”) to R98 “unattended death”. Both human coders are able to deduce that the subject probably fell down the stairs and use the ICD10 code W10 “Fall on and from steps”

| Text | cardiopathie ischemique avec triple pontage, anevrisme aortique, cancer de la vessie, hta, arret cardio - respiratoire |
| --- | --- |
| Predicted ICD10 | I259 Z951 I719 C679 I10 R092 |
| Database ICD10 | I259 I251 I719 C679 I10 R092 |
| Expert ICD10 | I259 I251 I719 C679 I10 R092 |

Table S5. Example of error caused by a coding rule. I251 and Z951 are both suitable for “triple pontage” (Coronary artery bypass surgery). However, the M4 mortality coding rule (Special instructions on surgery and other medical procedures) dictates the code choice

# Bibliography

[1] R. Sennrich, B. Haddow, and A. Birch, ‘Neural Machine Translation of Rare Words with Subword Units’, in *Proceedings of the 54th Annual Meeting of the Association for Computational Linguistics (Volume 1: Long Papers)*, Berlin, Germany, Aug. 2016, pp. 1715–1725, doi: 10.18653/v1/P16-1162.

[2] ‘tensorflow/models’, *GitHub*. https://github.com/tensorflow/models (accessed Jan. 22, 2020).

[3] ‘[1706.03762] Attention Is All You Need’. https://arxiv.org/abs/1706.03762 (accessed Feb. 04, 2019).

[4] L. Falissard *et al.*, ‘A deep artificial neural network based model for underlying cause of death prediction from death certificates’, *arXiv:1908.09712 [cs, stat]*, Aug. 2019, Accessed: Jan. 22, 2020. [Online]. Available: http://arxiv.org/abs/1908.09712.

[5] A. van den Oord *et al.*, ‘WaveNet: A Generative Model for Raw Audio’, *arXiv:1609.03499 [cs]*, Sep. 2016, Accessed: Jan. 29, 2021. [Online]. Available: http://arxiv.org/abs/1609.03499.
